# SPINK2 in hematopoiesis and cancer: Biology and clinical implications

**DOI:** 10.1038/s41420-026-03206-8

**Published:** 2026-07-10

**Authors:** L. Deligio, T. Loconte, AB Ventura, A. Negri, S. Ciavarella, G. Loseto, L. Viggiano, G. Castellano, G. Fiermonte, G. Volpe

**Affiliations:** 1 Hematology and Cell Therapy Unit, IRCCS Istituto Tumori “Giovanni Paolo II”, Bari, Italy; 2 Department of Biology, University of Bari “Aldo Moro”, 70125 Bari, Italy; 3 SJD Pediatric Cancer Center Barcelona, Institut de Recerca Sant Joan de Déu (IRSJD), Esplugues de Llobregat, Barcelona, Spain; 4 Department of Bioscience, Biotechnology and Environment, University of Bari “Aldo Moro”, 70125 Bari, Italy; 5 Department of Pharmacy – Pharmaceutical Sciences, University of Bari “Aldo Moro”, 70125 Bari, Italy

**Keywords:** Acute myeloid leukaemia, Mechanisms of disease

## Abstract

Serine protease inhibitors are essential regulators of tissue homeostasis, immunity, and cell survival, where they modulate tightly controlled proteolytic signaling networks that influence cell fate and microenvironmental interactions in diverse biological systems. Among them, Kazal-type inhibitors constitute a structurally distinct group defined by a compact, disulfide-rich domain that confers exceptional stability and potent inhibitory activity against a range of serine proteases. SPINK2, a relatively understudied member of this family, has recently gained increasing attention due to its emerging functional roles in haematopoiesis and hematological cancers, as well as for its known function in reproductive biology. Initially identified in the testis as a key regulator of protease-driven germ-cell maturation, *SPINK2* expression has since been found in bone marrow-derived hematopoietic stem and progenitor cells (HSPCs), where it contributes to the fine-tuning of protease-mediated signaling, cellular stress responses, and early lineage decisions. Growing evidence now implicates aberrant *SPINK2* expression in leukemogenesis, therapy resistance, and bone marrow failure syndromes, highlighting its potential importance as both a mechanistic determinant and a clinically relevant biomarker in hematologic disorders.

## Facts

- SPINK2 belongs to the family of Kazal-type serine protease inhibitors.
- *SPINK2* expression marks the emergence of the hematopoietic stem cells from the AGM region during gestation.
- *SPINK2* is highly expressed in acute myeloid leukemia.
- SPINK2 promote malignant features in leukemia and acts as a tumor suppressor in testicular carcinoma.

## Open questions

- What are the partners of SPINK2 that mediate its direct or indirect involvement in cancer?
- Can we pharmacologically manipulate SPINK2 to suppress leukemogenesis or lymphomagenesis?
- Why SPINK2 acts as a tumor suppressor in testicular cancer while promoting tumor establishment and progression in leukemia and lymphoma?
- Does SPINK2 act similarly in all hematological cancers?

## Introduction

SPINK2 (Serine Protease Inhibitor Kazal-Type 2) is a member of the Kazal-type serine protease inhibitor family, a group of structurally conserved proteins characterized by compact domains stabilized by intramolecular disulfide bonds [1, 2]. These inhibitors modulate tightly regulated proteolytic pathways involved in tissue homeostasis, cellular stress responses, and developmental processes. SPINK2 was first identified in the early 1990s in human testis, where researchers isolated a novel Kazal-domain containing transcript associated with germ-cell maturation and acrosomal biology [3, 4]. Initial functional studies revealed that SPINK2 acts as a potent inhibitor of trypsin-like serine proteases, helping to protect developing germ cells from protease-induced damage within the testicular microenvironment [5–7].

Although it was initially considered testis-specific, subsequent transcriptomic and proteomic analyses demonstrated that *SPINK2* expression extends beyond the reproductive system. In fact, several studies have reported *SPINK2* to be highly expressed in hematopoietic tissues, including bone marrow–derived hematopoietic stem and progenitor cells (HSPCs) [8]. Recent single cell-resolution studies have shown that *SPINK2* expression is transiently upregulated during embryonic hematopoiesis, this being particularly enhanced during the transition from hemogenic endothelium towards the first definitive HSCs [9–12]. This has positioned SPINK2 as a marker of early hematopoietic emergence and as a potential regulator of protease-mediated signaling pathways that are critical for stem cell specification, quiescence, and stress adaptation [9].

Beyond the normal development, SPINK2 has also sparked large interest in cancer biology. It is markedly overexpressed in several hematologic malignancies, with some of the highest levels observed in acute myeloid leukemia (AML), especially within immature leukemic stem–like cell fractions [13, 14]. Elevated *SPINK2* expression has also been reported in pediatric leukemias, where its expression correlates with therapy resistance and adverse clinical outcomes [15]. Additional studies suggest roles in other malignancies, including testicular cancer [16] and other solid tumors (e.g. hepatocellular carcinoma) [17, 18], although its mechanistic contributions in these contexts remain under investigation. Together, these findings underscore the emerging importance of SPINK2 as both a biological regulator and a potential disease-associated biomarker. However, it is important to emphasize that much of the current evidence supporting these roles is derived from expression-based and correlative studies, rather than direct mechanistic investigation. In particular, the distinction between association and causation remains insufficiently addressed in the literature, and further functional studies will be required to establish whether SPINK2 plays a direct regulatory role or primarily reflects underlying cellular states.

## SPINK family and the discovery of SPINK2

The serine protease inhibitor Kazal-type (SPINK) family comprises a group of small, secreted proteins unified by the presence of the Kazal domain, a compact inhibitory module of approximately 55–60 amino acids that blocks the active site of trypsin-like serine proteases. The Kazal fold, first recognized by Kazal and colleagues [1], is defined by a conserved cysteine-spacing pattern and a stable architecture consisting of a short alpha-helix, a three-stranded antiparallel beta-sheet, and a reactive site loop. The fold is stabilized by three intramolecular disulfide bonds arranged in the canonical Cys1–Cys5, Cys2–Cys4, and Cys3–Cys6 pattern, which confers exceptional proteolytic and thermal stability, enabling SPINK proteins to function in protease-rich or inflammatory environments [19, 20].

The founding member of the family, namely SPINK1, was discovered in the late 1940s during investigations into pancreatic autodigestion and was originally termed pancreatic secretory trypsin inhibitor (PSTI) by Kazal et al. [1]. Its discovery established the principle that endogenous protease inhibitors are essential for epithelial and tissue protection [21, 22]. Over subsequent decades, additional canonical SPINKs (SPINK1-SPINK9) were identified across diverse tissues, reflecting the functional broadening of SPINK biology from exocrine physiology to mucosal immunity, skin barrier homeostasis, wound repair, digestive protection, and reproductive biology [23, 24]. SPINK4 was linked to goblet cell function in the gastrointestinal tract [25, 26]; SPINK5 (LEKTI), with its 15 Kazal repeats, was shown to regulate kallikrein-driven epithelial desquamation [27, 28]; and SPINK6–SPINK9 were identified primarily in the aerodigestive tract and skin, where they modulate kallikrein activity and contribute to innate defense [29–37].

In addition to these nine canonical members, the human genome contains several SPINK-like paralogs, those being SPINK10, SPINK11, SPINK12, SPINK13 and SPINK14 [38–40]. These genes contain predicted Kazal-like domains but diverge substantially from SPINK1-SPINK9 in sequence conservation, cysteine spacing, and expression patterns. They are generally expressed at very low levels, often in testis or restricted epithelial contexts, and lack robust biochemical evidence of serine protease inhibitory activity [38]. For these reasons, they are considered SPINK-like genes belonging to the broader Kazal-domain superfamily rather than bona fide members of the functional SPINK family. Emerging evidence, however, suggests that some may have acquired specialized functions. SPINK13, for instance, has recently been reported to act as a tumor suppressor in hepatocellular carcinoma by modulating PTEN and inhibiting AKT phosphorylation, indicating that functional divergence does not necessarily imply biological inactivity [40]. A list of all SPINK family members, their function and expression patterns is provided in Table 1.

**Table 1:** List of SPINK family members, their expression patterns and function.

Gene	Status	Kazal Domain(s)	Tissue Expression	Known Function	Experimental Evidence
SPINK1	Canonical	1	Pancreas, duodenum, prostate	Trypsin inhibitor; pancreatitis protection	Strong biochemical + structural
SPINK2	Canonical	1	Testis, HSCs, AML	Protease inhibitor; roles in spermatogenesis, hematopoiesis, leukemia	Strong (functional assays, transcriptomics)
SPINK3 (rodent)	Not human	—	—	Mouse ortholog of human SPINK1	—
SPINK4	Canonical	1	Goblet cells, gut mucosa	Mucosal protection	Some functional data
SPINK5 (LEKTI)	Canonical	15 Kazal domains	Skin, thymus	Regulates kallikreins; barrier function; mutated in Netherton	Strong
SPINK6	Canonical	1	Nasopharynx, airway	Inhibits KLK5, KLK7, KLK14	Well-tested
SPINK7	Canonical	1	Esophagus	Anti-inflammatory; mucosal defense	Moderate
SPINK8	Canonical	1	Airway, GI tract	KLK regulation (predicted)	Low–moderate
SPINK9	Canonical	1	Skin	KLK activity regulation; wound response	Some functional
SPINK10	SPINK-like	1 (predicted)	Very low expression	Unknown; computationally annotated	No functional data
SPINK11	SPINK-like	1 (predicted)	Testis-biased low expression	Unknown	No functional data
SPINK12	SPINK-like	1 (predicted)	Testis/epididymis (weak)	Unknown	No functional data
SPINK13	SPINK-like	1 (predicted)	Brain/testis low expression	Unknown	No functional data
SPINK14	SPINK-like	1 (predicted)	Minimal expression	Unknown	No functional data

Within this broader gene family, SPINK2 represents a biologically distinctive member with unique roles in both germ-cell and hematopoietic biology [6, 10]. SPINK2 was discovered in the early 1990s during screens aimed at identifying testis-enriched transcripts involved in spermatogenesis. Its sequence encodes a single Kazal domain structurally analogous to that of SPINK1 [2, 41]. Early characterization localized SPINK2 to spermatocytes and spermatids, particularly within the acrosomal compartment, where it functions as an inhibitor of acrosin and related trypsin-like proteases [7, 42–48]. This role is crucial in preventing premature protease activation, which can damage developing germ cells during acrosome biogenesis and remodeling [7, 49, 50].

Subsequent high-throughput transcriptomic studies, however, revealed that *SPINK2* expression is not restricted to testis. It is markedly enriched in hematopoietic stem and progenitor cells (HSPCs), with single-cell RNA sequencing showing strong expression in primitive stem cell fractions [8, 10–12]. During embryogenesis, SPINK2 forms part of the transcriptional signature of nascent hematopoietic stem cells emerging from hemogenic endothelium, suggesting a conserved role in the regulation of protease activity within developmental stem-cell niches [9]. These findings imply that SPINK2 may help shape the extracellular proteolytic environment surrounding stem cells, possibly influencing quiescence, adhesion, and microenvironmental stability.

The recognition that *SPINK2* is expressed in HSPCs provided a foundation for understanding its emerging importance in hematologic malignancies. *SPINK2* is consistently upregulated in acute myeloid leukemia (AML), particularly within leukemic stem like cell populations [13, 14]. Elevated *SPINK2* correlates with poor prognosis, therapy resistance, and maintenance of stemness-associated transcriptional programs [15]. Mechanistic studies suggest that SPINK2 may support leukemic survival through multiple pathways, including buffering protease-mediated stress, modulating ferroptosis regulators, stabilizing extracellular matrix interactions, and influencing immune-related signaling [13, 51]. These insights point to SPINK2 as a potential mediator of microenvironmental protection in leukemia, analogous to its protective roles in germ cell biology.

Collectively, the chronological discovery and characterization of SPINK1 through SPINK9, together with the identification of SPINK-like paralogs, highlight the evolutionary versatility of the Kazal domain as a structural scaffold. Canonical SPINK proteins retain strong protease inhibitory activity and play well-established roles in epithelial protection, mucosal defense, barrier integrity, and reproductive physiology [31, 52, 53]. SPINK-like genes, while less conserved and generally unvalidated experimentally, may represent evolutionary experiments within the Kazal-domain superfamily, with emerging evidence supporting context-dependent roles for some members [39, 40, 54].

Within this landscape, SPINK2 occupies a distinctive position that bridges reproductive biology, hematopoietic development, and malignant transformation. Its conserved inhibitory activity, restricted expression pattern, and disease relevance set it apart from other SPINK proteins and underscore the importance of protease regulation as a unifying principle across both normal developmental processes and cancer biology. The ongoing study of SPINK2 continues to expand the conceptual boundaries of the SPINK family and reveals how a structurally simple inhibitory domain can be adapted to regulate complex cellular and microenvironmental processes.

## SPINK2 in hematopoiesis

**Fig. 1:**
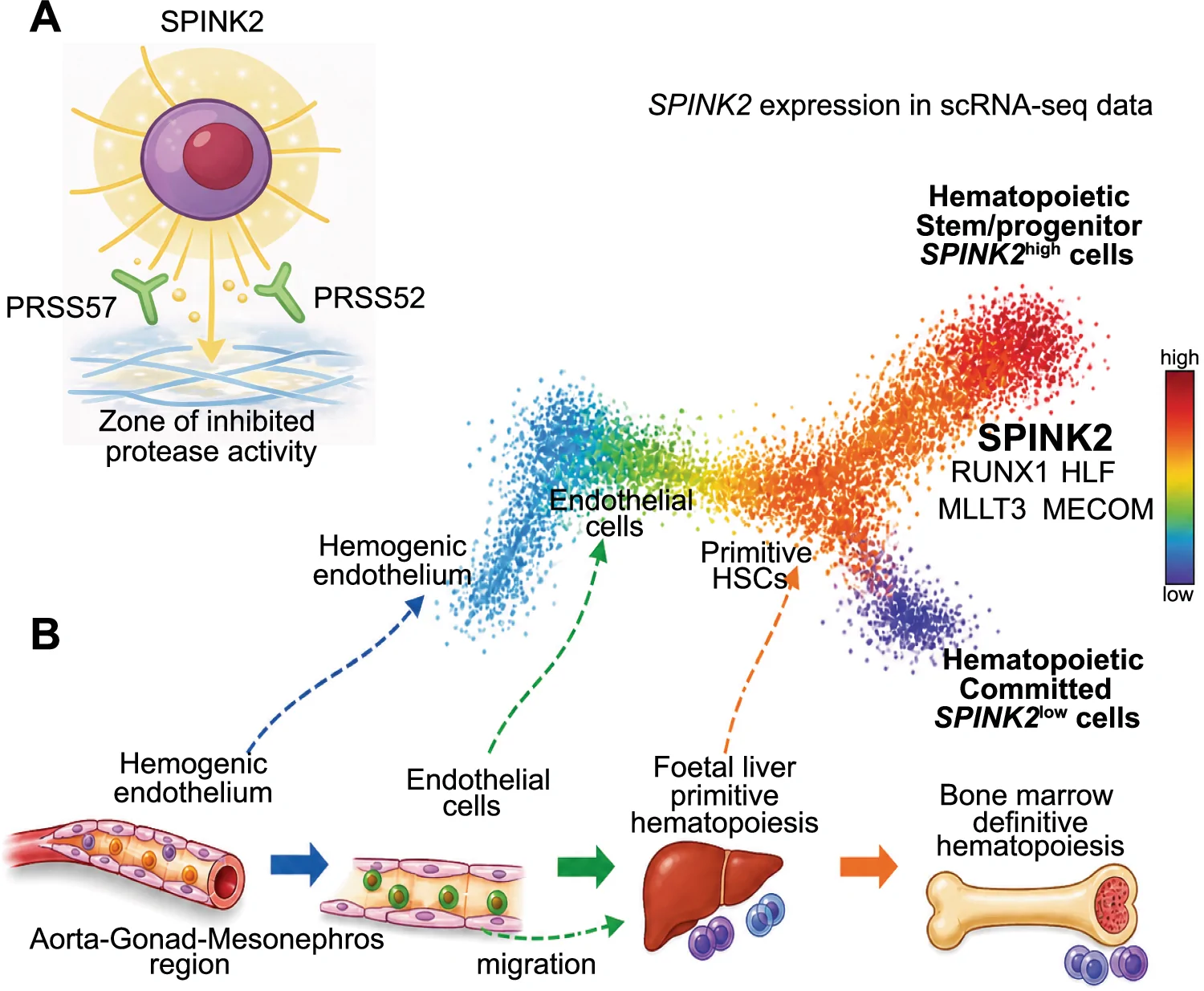
Schematic representation of SPINK2 expression and function during hematopoietic development. **A** Schematic representation of SPINK2 acting as a serine protease inhibitor that restricts protease activity (e.g., PRSS57 and PRSS52), creating a localized zone of inhibited protease activity. **B** Schematic illustration of *SPINK2* expression along the endothelial-to-hematopoietic transition and during hematopoietic development, from hemogenic endothelium in the aorta–gonad–mesonephros (AGM) region to fetal liver primitive hematopoiesis and adult bone marrow definitive hematopoiesis, resolved through single cell RNA-sequencing. *SPINK2* expression is enriched in hematopoietic stem/progenitor cells and co-expressed with stemness-associated transcription factors including *RUNX1*, *HLF*, *MLLT3*, and *MECOM*.

In normal hematopoiesis, SPINK2 emerges as a noteworthy albeit still not fully-characterized player within the haematopoietic stem/progenitor cell (HSPC) compartment. Single cell RNA-sequencing data from human adult bone marrow CD34⁺ cells demonstrate that *SPINK2* is highly expressed in the most primitive HSPC populations, those being the true hematopoietic stem cells (HSCs), multipotent progenitors, and common myeloid progenitors, where its mRNA abundance reaches the hundreds of counts-per-million and nearly 90-100% of cells in some subpopulations express *SPINK2* [10, 12, 55]. In contrast, *SPINK2* expression drops markedly in more differentiated progenitors (e.g., erythroid, lymphoid) and in mature myeloid/lymphoid lineages [55]. Functionally, SPINK2 has been shown to inhibit target serine proteases, and in particular those isoforms such as PRSS2 (trypsin-2) and PRSS57, via competitive, temporary enzyme inhibition kinetics [10]. PRSS57 is a serine protease expressed in hematopoietic cells and potentially involved in hematopoietic commitment, whereas PRSS2 belongs to the trypsin-like serine protease family involved in extracellular proteolysis and signaling [56, 57]. Beyond these, additional proteases may represent potential SPINK2 targets based on sequence similarity and expression patterns, although direct biochemical validation remains limited. Overall, the full spectrum of SPINK2 targets in hematopoiesis and their functional roles remain incompletely defined. However, these inhibitory interactions have been primarily demonstrated in biochemical and in vitro systems, and their physiological relevance within the bone marrow niche remains to be fully validated in vivo. Through mathematical modeling of this activity, a “zone of inhibited protease activity” surrounding SPINK2-secreting HSPCs was proposed, suggesting that SPINK2 might modulate the extracellular proteolytic microenvironment of stem cells within the bone marrow niche [10] (Fig. 1A). This model provides a useful conceptual framework; however, it is based on computational and indirect evidence rather than direct experimental validation in vivo, and should therefore be interpreted with caution. Such regulation may help maintain stem cell quiescence or protect against excessive protease-mediated degradation of the niche or mobilizing matrix components. Moreover, developmental studies of human embryonic hematopoiesis show that *SPINK2* is also part of the transcriptional signature of nascent HSCs emerging from hemogenic endothelium (in combination with genes such as *RUNX1*, *MECOM*, *MLLT3* and *HLF*) during the aorta-gonad-mesonephros (AGM) to liver colonization wave [9] (Fig. 1B). Altogether, these observations indicate that SPINK2 is not simply a passive marker but may play a functional role in maintaining HSC identity and in regulating stem-cell-niche interactions during ontogeny and in the adult.

## The function of SPINK2 in hematological cancers

SPINK2, a member of the serine protease inhibitor (Kazal-type) family, has amassed growing attention in hematologic malignancies because of its aberrant expression patterns and associations with poor prognosis, therapy resistance, and stem cell-like features of malignant blasts.

A first indication of SPINK2 involvement in hematological disorders emerged from an early and influential study by Hoefnagel and colleagues [58]. In this work, the authors employed cDNA microarray-based transcriptional profiling to identify molecular determinants capable of distinguishing primary cutaneous large B-cell lymphoma of the leg from primary cutaneous follicle center cell lymphomas [58, 59]. Notably, they reported that the latter subgroup exhibited markedly elevated *SPINK2* expression (approximately a 17-fold increase) relative to the former. Despite this intriguing observation, no subsequent investigations have explored SPINK2 in this disease subtype, leaving its potential functional or mechanistic contribution within these specific lymphoma contexts largely unexplored and poorly understood.

With respect to leukemia, a lot more studies are available and have emerged in the last few years, demonstrating elevated *SPINK2* mRNA in AML patients relative to healthy controls [14, 51, 60]. Prognostically, higher *SPINK2* expression has been associated with reduced overall survival, increased relapse risk, and induction therapy failure in several disease cohorts [15, 55].

More recently, SPINK2 protein expression was shown to be an independent adverse biomarker in AML: in a cohort study it correlated with intermediate cytogenetic risk, NPM1 mutation status, and European LeukemiaNet (ELN) 2022 risk classification, and helped refine prognostic stratification beyond standard risk factors [51]. At the functional level, transcriptomic analyses in AML reveal that high *SPINK2* associates with gene-sets corresponding to ferroptosis regulation, immune response modulation, and pathways such as PI3K-AKT and PD-L1 immune-checkpoint signaling. These associations are largely derived from gene expression analyses and do not establish direct causal relationships between SPINK2 activity and these pathways. In the context of ferroptosis, functional studies suggest a more direct mechanistic link, as *SPINK2* knock-down modulates the expression of key regulators such as *SLC7A11* and *STEAP3*, alters cystine uptake and intracellular iron levels, and increases sensitivity to erastin, a small molecule inhibitor that induces ferroptotic cell death [51]. These observations indicate that SPINK2 may influence cellular redox balance and glutathione metabolism, thereby limiting lipid peroxidation and protecting leukemic cells from ferroptotic cell death. By contrast, the association with PI3K-AKT signaling is less well defined mechanistically. A plausible hypothesis is that SPINK2, through its protease inhibitory activity, may modulate the extracellular proteolytic environment, thereby influencing the availability or activation of growth factors and their receptors, which in turn could impact downstream survival pathways. However, this link remains to be experimentally validated. In parallel, *SPINK2* inhibition up-regulates ALCAM (a T-cell-activating molecule) suggesting immune environment effects. This observation supports a potential role for SPINK2 in modulating leukemic cell–immune interactions. In this context, the association with PD-L1-related pathways may reflect an indirect contribution to immune evasion, possibly through regulation of immune-interacting molecules or microenvironmental signaling, although a direct mechanistic connection between SPINK2 and immune checkpoint regulation has not yet been established.

**Fig. 2:**
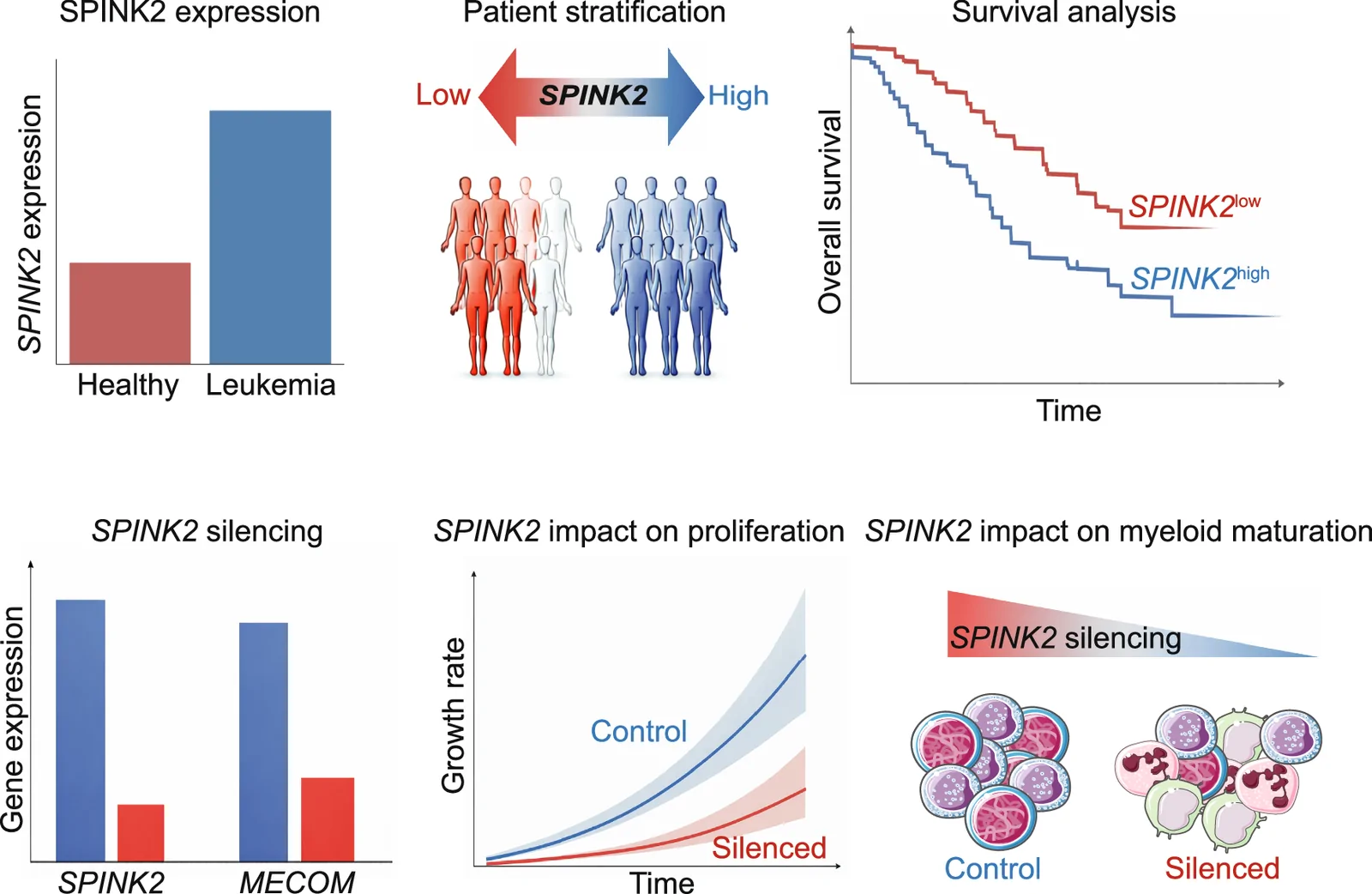
Schematic representation of *SPINK2* expression and relevance in acute myeloid leukemia (AML). *SPINK2* expression is elevated in AML compared to healthy individuals. When AML patients are stratified according to *SPINK2* expression levels, those with high expression levels exhibit significantly worse overall survival compared with patients with low *SPINK2* expression. Functional studies indicate that *SPINK2* silencing reduces the expression of *SPINK2* and the stemness-associated transcription factor *MECOM*, leading to decreases leukemic cell proliferation while promoting terminal myeloid maturation.

Mechanistically, while causality remains unclear, several hypotheses exist. Notably, most supporting evidence comes from in vitro models, with limited in vivo data demonstrating SPINK2’s role in leukemogenesis or disease progression. One is that SPINK2, by inhibiting serine proteases, may support leukemic stem/progenitor cells (LSCs) to maintain a protective niche micro-environment and resist differentiation or death [61]. This view is consistent with scRNA-seq data showing that AML blasts with HSC-like phenotypes preferentially express *SPINK2* together with HSC-associated genes, including *MYB*, *HOXA9* and *GFI1* [55, 62–65]. Another mechanism is that SPINK2’s influence on ferroptosis may grant AML cells a survival advantage under oxidative stress or therapeutic challenge; by limiting ferroptotic death, blasts may become more resilient to induction therapy. Immune evasion is likely a third facet: by lowering expression of immune-activating molecules and modulating checkpoint pathways, cells with high *SPINK2* may attenuate host immune responses. Recent functional work has begun to directly address the biological role of *SPINK2* in AML. In particular, in a study conducted in our lab, silencing of *SPINK2* in leukemic cell models significantly reduced proliferative capacity and impaired clonogenic growth, while promoting transcriptional programs associated with myeloid differentiation. Mechanistically, *SPINK2* depletion resulted in downregulation of *MECOM*, a transcription factor strongly associated with aggressive AML, leukemic stem cell maintenance, and poor clinical outcome. MECOM is a key regulator of hematopoietic stemness programs and has been implicated in maintaining self-renewal capacity with blocking myeloid differentiation in AML [66, 67]. Consistent with this regulatory link, suppression of the *SPINK2- MECOM* axis was accompanied by partial restoration of myeloid lineage commitment and attenuation of leukemic self-renewal programs [55] (Fig. 2). These findings suggest that SPINK2 may contribute to the maintenance of leukemic stem-like states through modulation of transcriptional programs downstream of MECOM, thereby reinforcing proliferation and blocking differentiation. These findings provide experimental evidence that SPINK2 contributes not only to AML prognosis but also to the maintenance of the leukemic state, supporting a model in which SPINK2 acts upstream of transcriptional networks that sustain proliferation and block differentiation in AML blasts. Within the broader context of AML biology, this axis places SPINK2 upstream of a well-established stemness-associated regulatory node, linking extracellular protease regulation to transcriptional control of leukemic cell fate.

From a translational perspective, SPINK2 holds potential both as a biomarker and therapeutic target in hematologic cancer. Its expression could refine risk stratification and perhaps guide treatment intensity or novel therapeutic choice. The identification of small-molecule inhibitors of SPINK2 in preclinical work further raises the possibility of targeted disruption of SPINK2 function in high-risk AML. However, key gaps remain, including the need for direct functional studies (in vivo and in vitro) on SPINK2 loss/gain in leukemic cells, the identification of its precise protease targets in leukemic context, and the determination of whether SPINK2 inhibition can sensitize cells to standard therapies or overcome resistance.

In summary, SPINK2 emerges as a multifaceted mediator in hematologic malignancies, most notably in AML, linking stem-cell like biology, ferroptosis resistance, and immune evasion into a combined axis of malignancy maintenance [15, 55, 61]. These effects are likely mediated through a combination of direct protease inhibition and indirect modulation of metabolic, signaling, and immune pathways, although the precise molecular connections remain to be fully elucidated. Deepening our understanding of SPINK2’s roles may unlock new prognostic tools and therapeutic avenues in aggressive hematologic cancers.

## The involvement of SPINK2 in solid cancers

Although SPINK2 is best studied in hematologic malignancies, emerging evidence indicates that it also plays biologically meaningful and context-dependent roles in several solid cancers. Unlike in acute myeloid leukemia, where SPINK2 typically functions as an oncogenic and prognostically adverse molecule, studies in solid tumours increasingly suggest that SPINK2 may behave as a tumour suppressor, particularly in epithelial and germ-cell derived malignancies. One of the most detailed characterizations comes from testicular cancer, where SPINK2 expression is markedly reduced in tumour tissue compared to normal testis. Mechanistic studies in testicular carcinoma cell lines have shown that SPINK2 interacts directly with TIG1 (also known as RARRES1), forming a protein complex that suppresses epithelial-mesenchymal transition and reduces cell migration and invasion [16]. This suppression is mediated through inhibition of the uPA/uPAR signaling axis, a pathway strongly implicated in extracellular matrix degradation, metastasis, and tumour invasiveness. Loss of SPINK2 therefore removes a critical layer of protease regulation that ordinarily restrains malignant progression of testicular germ-cell tumours [16].

A similar tumour-suppressive pattern has been observed in hepatocellular carcinoma (HCC). Several studies report decreased SPINK2 expression in HCC tissues relative to healthy liver, and functional experiments reveal that restoring SPINK2 in HCC cell lines slows proliferation, reduces migratory capacity, and sensitizes cancer cells to targeted therapies such as Lenvatinib [16, 18]. These findings suggest that SPINK2 contributes to maintaining protease homeostasis and may antagonize oncogenic pathways driving hepatic tumour growth [17]. The observation that SPINK2 enhances sensitivity to lenvatinib further raises the possibility that SPINK2 could be used as a predictive biomarker to guide therapeutic decision-making or to stratify patients who might benefit from specific kinase inhibitors [18].

Beyond testicular cancer and HCC, transcriptomic surveys indicate that *SPINK2* expression is detectable at low levels across several solid cancer types, though its functional significance in most of these contexts remains largely unexplored. Given that SPINK family proteins are classical secreted protease inhibitors involved in maintaining epithelial integrity [68], it is plausible that SPINK2 contributes to tissue-specific control of proteolytic balance, extracellular signaling, and interactions with the tumour microenvironment. Loss of such regulatory functions could therefore promote invasion or metastatic potential in certain epithelial cancers, as seen with other SPINK family members [52, 69–71].

Overall, current evidence portrays SPINK2 as a potentially protective factor in solid malignancies, with down-regulation contributing to tumour progression (see Table 2). First, tissue-specific protease landscapes may dictate SPINK2 activity, as target proteases differ between hematopoietic and epithelial contexts. Second, microenvironmental differences may contribute, with SPINK2 supporting leukemic stem cell survival in the bone marrow while maintaining epithelial integrity in solid tissues. Third, context-specific interaction partners, such as TIG1/RARRES1 in testicular cancer, may redirect SPINK2 toward tumor-suppressive functions. Finally, variations in cellular differentiation state and stress responses, including ferroptosis susceptibility, may further influence these divergent effects. Further mechanistic studies are needed to clarify how SPINK2 integrates into protease networks and signaling pathways across different tissue types, and whether restoring SPINK2 activity may offer therapeutic benefit in selected solid cancers.

**Table 2:** Overview of SPINK2 expression and function across different cancer types.

Cancer type	Expression vs normal	Functional role	Mechanistic axis	Key evidence	Clinical relevance
AML	High ↑↑	Oncogenic	Ferroptosis resistance, stemness, immune evasion	Transcriptomics + knockdown studies; Xue et al. 2019; Chen et al. 2022	Prognostic biomarker, potential target
ALL (pediatric)	High ↑	Oncogenic	Therapy resistance	RNA-seq studies; Barresi et al., 2021	Risk stratification
Cutaneous B-cell lymphoma	High ↑	Unknown	—	Microarray studies; Hoefnagel et al., 2005	Not followed up
Testicular cancer	Low ↓	Tumour-suppressive	TIG1 interaction, uPA/uPAR suppression	Functional assays; Shyu et al., 2019	Metastasis suppressor role
Hepatocellular carcinoma (HCC)	Low ↓	Tumour-suppressive	ECM regulation, lenvatinib senitization	Overexpression studies; Guo et al., 2024	Predictive biomarker potential

## Pharmacological and therapeutic targeting of SPINK2

Despite the growing recognition of SPINK2 as a functionally important mediator in several malignancies and most notably acute myeloid leukemia (AML), the pharmacological targeting of SPINK2 remains in the earliest stages of investigation. SPINK2 plays key roles in regulating protease activity, ferroptosis signaling, immune modulation, and stem-cell–like transcriptional programs in leukemic blasts [51]. Its high expression in AML and association with chemoresistance and poor prognosis have prompted interest in developing therapeutic strategies that disrupt SPINK2 function or expression. The most direct evidence for pharmacological tractability comes from a recent AML study in which researchers identified a potential small-molecule inhibitor (SMI) of SPINK2 [51]. Although the compound remains unnamed and uncharacterized in detail, the authors highlight its ability to modulate SPINK2-associated pathways. However, this evidence should be considered early-stage and proof-of-concept, as it is based on a single small-molecule inhibitor and lacks comprehensive pharmacological and in vivo validation. This study therefore represents a preliminary step toward the pharmacological targeting of SPINK2 in hematologic cancer.

Beyond this initial lead, broader literature on the SPINK protein family indicates that SPINKs are increasingly viewed as therapeutic targets in cancer, with several reviews discussing their involvement in protease-regulated tumour progression, immune biology, and tissue remodeling [53, 72]. In this context, SPINK2 is frequently highlighted as a potential target, particularly in malignancies where it is aberrantly overexpressed. Although these reviews do not provide specific drug candidates for SPINK2 inhibition, they frame it within a family of proteins for which biologic or small-molecule modulation is conceptually feasible.

A complementary and technically significant development is the engineering of SPINK2-based scaffold proteins with high affinity and specificity for target proteases. These engineered SPINK2 variants have been used as customizable binding scaffolds, demonstrating that the structural backbone of SPINK2 can be manipulated to produce highly stable inhibitor molecules [73, 74]. While this work does not directly generate therapeutics against SPINK2, it showcases the biochemical malleability of the protein and underscores the feasibility of designing molecules that can disrupt protease-SPINK2 interactions or compete with endogenous SPINK2 binding partners.

Together, these studies suggest that pharmacological targeting of SPINK2 is biologically plausible but underdeveloped. The identification of a preliminary SMI in AML provides a foundational proof-of-concept, while the expanding understanding of SPINK2’s functional roles offer multiple mechanistic rationales for therapeutic intervention. Future efforts will require structural studies of SPINK2, delineation of its protease targets in malignant and normal contexts, and preclinical testing of both small-molecule inhibitors and biologics capable of modulating SPINK2 activity. Given its restricted expression pattern in normal hematopoiesis and its strong association with disease aggressiveness, SPINK2 represents a compelling, though still largely untapped, therapeutic target in hematologic malignancies.

## Conclusion

Together, the emerging literature positions SPINK2 as a multifaceted regulator in both normal and malignant hematopoiesis, with context-dependent roles extending into select solid tumours. In healthy development, SPINK2 contributes to the proteolytic microenvironment that supports HSC identity and niche interactions, while in hematologic cancers and in particular in AML it becomes aberrantly upregulated, promoting ferroptosis resistance, immune evasion, and maintenance of leukemic stem-cell–like features. Conversely, in several solid cancers, SPINK2 appears to function more as a tumour suppressor, restraining invasion, EMT, or proliferation when expressed. Although pharmacological targeting of SPINK2 is still nascent, early identification of small-molecule inhibitors and the demonstrated engineerability of SPINK2-based scaffolds suggest growing therapeutic potential. Continued mechanistic dissection of SPINK2’s protease targets, signalling partners, and niche functions will be essential for translating these insights into novel biomarkers or targeted therapies across cancer types. In particular, major outstanding questions include the identification of bona fide SPINK2 protease targets in physiological and malignant contexts, the mechanistic basis of its context-dependent oncogenic versus tumor-suppressive roles, and the feasibility of therapeutic targeting in vivo. Addressing these challenges will be critical to fully define the biological and clinical relevance of SPINK2.
